# An exploration of the role of trust and rapport in enhancing vaccine uptake among Anishinaabe in rural northern Ontario

**DOI:** 10.1371/journal.pone.0308876

**Published:** 2024-12-05

**Authors:** Chris Sanders, Kristin Burnett, Lana Ray, Marina Ulanova, Donna M. Halperin, Scott A. Halperin, on behalf of the Canadian Immunization Research Network

**Affiliations:** 1 Department of Sociology, Lakehead University, Thunder Bay, Ontario, Canada; 2 Department of Indigenous Learning, Lakehead University, Thunder Bay, Ontario, Canada; 3 School of Nursing, Lakehead University, Thunder Bay, Ontario, Canada; 4 NOSM University, Thunder Bay, Ontario, Canada; 5 Elizabeth & Thomas Rankin School of Nursing, St. Francis Xavier University, Antigonish, Nova Scotia, Canada; 6 Division of Infectious Diseases, IWK Health Centre, Dalhousie University, Halifax, Nova Scotia, Canada; University of Greenwich, UNITED KINGDOM OF GREAT BRITAIN AND NORTHERN IRELAND

## Abstract

This article examines the complicated terrain of immunization acceptance and access among Indigenous peoples in northern Ontario by drawing on conversations held prior to 2019 that explored knowledge about *Haemophilus influenzae* type a (Hia) infection specifically and attitudes toward vaccines more broadly. In the decade preceding COVID-19, Hia emerged as a leading cause of morbidity and mortality in Indigenous communities in northern Canada. Before developing new vaccines, it is imperative to hold conversations with the communities most affected and to learn more about Indigenous peoples’ perceptions of and knowledge about vaccines, both generally and Hia specifically. We conducted focus groups and one-on-one conversations with Anishinaabe Peoples in northwestern Ontario. Our findings illustrate that decisions to vaccinate are informed by a host of social, institutional, and ideological factors and historical and contemporary relationships with government institutions and health practitioners. In particular, Indigenous community members perceived their relationships with social and health services and education institutions as coercive. Thus, public health approaches cannot continue to operate in ways that prioritize interventions for Indigenous peoples and communities so that they “do the right thing.” More emphasis is needed on health service and social service provider knowledge, skills, attitudes and practices-redirecting the onus onto those within the health care system. Solutions must respect Indigenous nationhood and the right of self-determination. Finally, we suggest the term vaccine hesitancy may not entirely capture the breadth of experiences that many Indigenous Peoples and communities have and continue to have within the health care system in Canada.

## Introduction

A delay or refusal of vaccination for infectious or non-communicable disease despite its availability has been coined “vaccine hesitancy” by the Strategic Advisory Group of Experts on Immunization (SAGE) [1]. Concerns about the pervasiveness of vaccine hesitancy have grown in North America over the last decade in response to outbreaks of contagious childhood diseases like measles and pertussis [2–4], and especially in the context of the COVID-19 global pandemic [5]. In 2019, the World Health Organization identified vaccine hesitancy as one of the ten threats to global health [1]. As a result, there is growing interest in individuals and groups who are perceived to be “vaccine hesitant” [6], and a number of explanatory models have been proposed, like the 3C and, more recently, the 5C model for COVID-19. For instance, the 5C model identified five characteristics that frequently inform the choices individuals make about vaccines: confidence or trust in public health science, vaccine safety, and effectiveness; complacency or perceived level of threat of the disease; constraints or structural and psychological barriers, including political and sociocultural barriers and psychological distress related to vaccination intention and uptake; risk calculation or the comparison of risks of infection versus vaccination; and, collective responsibility or becoming vaccinated to protect others and generate herd immunity [7]. The existence of a multitude of explanatory models suggests that there is not one specific reason why individuals delay or refuse vaccines. Indeed, decisions to vaccinate are informed by a host of social, institutional, and ideological factors [8, 9] and historical and contemporary relationships with government institutions and health practitioners [10–12].

Unfortunately, the label of vaccine hesitancy tends to be applied uniformly and all variables used to explain why people delay or refuse vaccines, including mistrust and systemic racism, are included under the large umbrella of “vaccine hesitancy,” thus obscuring more than it reveals. Research on vaccine hesitancy among Indigenous and racialized populations illustrates that historical and ongoing experiences with medical violence [13], which are a function of settler colonialism, systemic racism, and a lack of personal choice, fundamentally inform the choices people make about immunization [10, 12, 14–16]. Over the last two centuries the Canadian state created settler colonial systems of governmentality intended to erase the social, political, and economic existence of Indigenous peoples in the geographic area that came to be known as Canada. Residential schools represent perhaps one of the most brutal examples of settler colonial violence in Canada. Starting in the late 19^th^ century, residential schools were carceral institutions run by the federal government in partnership with Churches, whose sole objective was to separate Indigenous children from their families in order to assimilate them [17]. Parents who refused to send their children to residential schools faced severe financial penalties and jail time. The schools frequently served as laboratories for medical experimentation. For example, from 1942–1952 the federal government, along with leading medical researchers in Canada, performed nutrition experiments on Indigenous children in six residential schools, in some cases withholding food and medical assistance in order to explore the impact of malnutrition on the development of children [18]. It is in this manner (and many others) that the schools served as extremely violent institutions that sexually, physically, and emotionally harmed Indigenous children. Thus, enfolding centuries of medical mistreatment, violence, and coercion into the broad category of vaccine hesitancy reduces the distinct experiences of Indigenous peoples and communities [19, 20].

While important as a starting point to understanding vaccine hesitancy generally, models like 5C cannot adequately account for the long histories of epistemological and cultural violence that have taken place under settler colonialism, where we witnessed the systematic destruction, criminalization, and denigration of Indigenous systems of knowledge through genocidal state apparatuses like the Indian Act and residential schools [10]. As Greenwood and MacDonald persuasively argue “enhancing vaccine acceptance among Indigenous peoples will rest on pairing messaging about the scientific efficacy of the COVID-19 vaccine with information that is grounded in the strengths and wisdoms of [Indigenous] teachings.” [10]. Settler colonialism and systemic racism cannot be reduced to variables under the broader category of vaccine hesitancy. Rather, we must see them as a defining feature of Indigenous peoples’ experiences and perceptions of public health practices in Canada.

At the beginning of the COVID-19 pandemic and during the early stages of the vaccine rollout, the potential for vaccine hesitancy among Indigenous peoples in Canada was identified as a possible concern [21]. Significantly, there was a lack of specificity in public health policy and planning, despite the generation of explanatory models, regarding the distinct reasons why Indigenous peoples in Canada may delay or refuse vaccines. Importantly, there were almost no conversations about the appropriateness of applying the term vaccine hesitant to Indigenous peoples. What some may identify as vaccine hesitant attitudes among Indigenous peoples is more accurately a combination of a systematic lack of access to necessary health services and infrastructure (including vaccine supplies) [22]; racism and distrust that has been produced and persists through government-created colonial health structures [6, 11, 23–25]; medical experimentation, forced sterilization and historical use of biological warfare against Indigenous peoples [10–12]; lack of representation in clinical trials and public health preparedness [24]; and preference for Traditional healing [12]. While these constraints are acknowledged to an extent under the 5C model, placing everything and everyone within the same explanatory model limits and erases the distinct experiences of Indigenous peoples within settler colonialism. Thus, we wonder if vaccine hesitancy is the best term for describing Indigenous peoples who delay or do not get vaccinated. When considering the development of new vaccines, the generation of immunization policies, and public health planning, it is necessary to understand Indigenous peoples’ relationships with the Canadian government and biomedicine, the historical and ongoing role played by settler colonialism, and Indigenous nationhood and the right of self-determination [10]. This article describes the complicated terrain of immunization acceptance and access among Indigenous peoples by drawing on conversations held prior to 2019 that explored knowledge about Hia specifically and attitudes toward vaccines more broadly.

Invasive *Haemophilus influenzae* type a (Hia) infection emerged, in the decade prior to the beginning of the COVID-19 global pandemic, as a leading cause of morbidity and mortality in Anishinaabe communities in northern Canada [26, 27]. Hia is a particularly pernicious disease, with most cases occurring in young children, with manifestations including meningitis, septicemia, septic arthritis, and bacteremic pneumonia [28, 29]. Incidence of invasive Hia disease had reached rates similar to those of invasive *H*. *influenzae* type b (Hib) disease before the introduction of the publicly funded conjugate Hib vaccine [30]. A candidate vaccine against Hia is under development in Canada [31, 32]. Although our research was carried out with Anishinaabe communities prior to COVID-19, and more research on perceptions and decision making regarding COVID-19 and immunization specifically are needed, our findings reveal that relationships of trust play an important role in addressing community concerns and serve as an illustrative example for non-Indigenous health care providers and policy makers.

We report on findings that emphasize the need to actively adopt anti-racism practices at all stages of medical research and health care systems and the importance of building rapport and trust in relationships between Indigenous peoples, Canadian government agencies, and Western health care providers. In the discussion section, we highlight the work in northwestern Ontario regarding COVID-19 vaccines undertaken by Anishinaabe leaders who engaged with community members to ensure concerns and questions were answered and to show by example the need to get vaccinated. We then discuss how the findings speak to broader historical and systemic issues that need to be addressed to improve health care access for Indigenous peoples and advance public health objectives for monitoring immunization schedules in rural and northern communities in Canada. Indeed, given that the Chief Medical Officer of Health for Indigenous Services Canada noted that when vaccine programs were first rolled out in early 2021, rates of vaccine uptake initially were four times higher in First Nations communities than in the general population, it would be to the benefit of non-Indigenous health care practitioners and the state to follow the example set by Anishinaabe leaders in northwestern Ontario who were instrumental to its success [33]. In short, conversations with Indigenous community members revealed priorities regarding the relationship between health and well-being and access to and use of vaccines and the health care system more broadly as being distinct from those proposed by non-Indigenous people, predominantly white researchers and scientists. Instead of viewing vaccines as a standalone issue, health care providers and policy makers need to look at a web of systems/relationships that include and connect racism, settler colonialism, and their operation and rootedness in Canada’s health care system alongside immunization policies and practice [34]. One cannot be addressed without the other.

## Methods

### Research approach and community engagement strategy

This study originated from a desire for settler biomedical researchers to better understand the knowledge of both Indigenous community members and health care providers in northern Ontario about Hia specifically and perceptions of vaccines more generally so that this information could inform their practice and the potential development of a vaccine. In 2016, biomedical researchers from the Canadian Immunization Research Network (CIRN) contacted two white settler scholars in the social sciences, with a history of working with Indigenous communities and organizations in northern Ontario, to carry out these conversations with First Nations members and communities. We also drew on the expertise of an Anishinaabe scholar to help interpret our data, and ensure that Indigenous perspectives from this region were centred in the analysis. Findings were shared and commented on by participants to ensure we represented their perspectives accurately. CIRN researchers felt it was extremely important to hold such conversations in order to ensure that Indigenous peoples could inform immunization policy and practice from its inception, as well as to educate biomedical scholars. This was especially important given the history of medical violence and experimentation experienced by Indigenous communities [35, 36]. However, it should be noted that while such preliminary conversations were and are essential, they do not replace and were not intended to replace actual engagement with Indigenous communities by biomedical researchers when moving forward with clinical trials.

Our methodology and community engagement acknowledges that the Indigenous population in northern Ontario is diverse and occupies multiple and intersecting geographic locales that include both rural and urban spaces [34]. For instance, by 2006 more than half of the individuals who identify as Indigenous in Canada lived in cities [37], with Thunder Bay having the largest per capita Indigenous population at 12.7 percent [38]—a number that is likely much higher. A recent study entitled “Our Health Counts” found that the actual number is closer to 2–4 times more than reported by Statistics Canada [39]. People also frequently move across their territories between First Nations and urban spaces like Thunder Bay for a variety of reasons [40], including to access health services. This diversity of experiences also extends to status (as recognized under the Indian Act) and the relationships Indigenous peoples have with their communities: some people identify as status Indians (i.e., they have legal status under the Indian Act) but are not members of a First Nation (i.e., they do not appear on a particular band’s membership list); others identify as both status and band members but have never lived in their First Nation (i.e., their parents may be band members, but reside in another location); and some identify as status or non-status people and live on reserve (i.e., married to someone who lives on reserve). In understanding how, why, and when people seek access to health care, we need to recognize how these services and relationships with providers are shaped by rural and northern spaces, as well as the strategies people employ [34].

As a result, our data collected included speaking with people who came from broad geographic and social locations and who possessed varying relationships with First Nations and formal Indigenous political structures. We worked with community research assistants who had experience with recruitment, data collection, and analysis. The research assistants and KB drew on their regional social networks to recruit people on and off-reserve. Using convenience and snowball methods, participants were approached through various networks in person and using flyers: social media, Chief and Council, friendship networks, and service organizations. Focus groups were conducted in community settings (e.g., coffee shops with booths or rooms for privacy, health centres including hospitals and nursing stations, schools, community centres, and band offices). As researchers, we needed to be confident that our research respected both Indigenous sovereignty and the choice of individuals to speak about and from their own experiences and perspectives [41]. We sought out and received permission from the Chief and Council to hold conversations on reserve because we were in their jurisdiction, however it was not appropriate to seek Chief and Council approval to speak to band members off-reserve as this would infringe on individual freedom. This diverse sampling approach ensured that we were reflective and responsive to the current realities of Indigenous peoples in northwestern Ontario and respectful of collective and individual autonomy and rights. The following principle, as outlined in the Tri-Council Policy Statement: Ethical Conduct for Research Involving Humans (TCPS2) [42] under Article 9.2 guided our engagement:

First Nations, Inuit and Métis persons, whether or not they identify as members of an Indigenous community, enjoy freedom of expression, as does any citizen. They are free to consent and to participate in research projects that they consider to be of personal or social benefit. If the project is unlikely to affect the welfare of the individuals’ communities, local community engagement is not required under this Policy.

(Nature and Extent of Community Engagement, point 5)

We employed a community based participatory research approach that was guided by participants to ensure our relationship was ethical and non-exploitive. As such, the research process was flexible and adaptable. Preliminary conversations with Indigenous peoples living in Thunder Bay began with a broad set of questions about Hia specifically and vaccines generally. As our research proceeded, we adjusted and reassessed project objectives. For instance, while we began our interviews with a broad set of questions generated in conjunction with our community research assistants, the direction and flow of the conversations we had were propelled by the participants. We agree with Martin’s call for research that reflects the perspectives of Indigenous communities in that it is predicated on “more than just the production of new knowledge, but upholds the pedagogical, political, moral and ethical principles that resist oppression and contribute to strategies that reposition research” [43]. This vision guided our approach as conversations shifted to focus on vaccines as part of a broader web of relationships and experiences in a settler health care system that are historical and ongoing in Canada.

Our research was also informed by Eve Tuck’s call to action in “Suspending Damage: A Letter to Communities,” where she challenges researchers to stop “documenting damage-centered research—research that intends to document people’s pain and brokenness to hold those in power accountable for their oppression” [44]. Guided by conversations with community members, we focused on the failures of the settler health care system and its dysfunction and violence towards Indigenous peoples. Participants requested that we use the information they shared with us to (re)educate and unsettle non-Indigenous researchers. Indeed, discussions of racism, a lack of respect, and the failure of non-Indigenous health care workers to build trust was not information unknown to participants. Instead, much like the cases of Joyce Echaquan and Brian Sinclair, such experiences were all too familiar and frequent [45, 46]. The themes were consistent across all focus groups and interviews.

### Individual interviews and focus group procedures

Data were collected between January 2018 and January 2020. Participants completed an anonymous questionnaire to gather demographic information (e.g., age, gender). Ten focus group conversations (72 participants) were conducted with community members, ranging in size from 3 to 11 people. Focus group participants ranged in age from 24–69 years. They were primarily female but were evenly distributed with regard to age and representation from young parents, single adults, and Elders. We developed an interview guide for focus groups in consultation with our community researchers [47]. The guide was revised to reflect the interests/concerns of participants. Focus groups were conducted by two researchers (CS, KB) with the assistance of community research assistants, who facilitated discussions and encouraged participation.

We also conducted individual interviews with 14 healthcare service providers who came from both Indigenous and settler backgrounds. Questions were organized around knowledge, beliefs, values, provider expertise and practices regarding Hia, vaccines in general, and experiences providing immunizations in northern and rural communities. Individual interviews with healthcare service providers were carried out by two researchers (CS, KB). Providers included physicians, nurses working at community health centers and public health units, and community health educators. Individual interviews took place in community settings and workplace offices; some practitioners in remote communities were interviewed by phone.

A similar informed consent process was performed for both individual interviews and focus group interviews (e.g., purpose of study, review of risks and benefits, volunteerism, answering questions). Individual interviews with healthcare service providers lasted between 45 to 60 minutes, while focus group interviews with community members lasted between 90 to 120 minutes. All individual interviews and focus group interviews were audio-recorded and transcribed verbatim. After data collection, we followed up with individual participants to verify our findings and share the results of our study. Additionally, we met with our partners at CIRN to share results and publish findings to make sure they were informed about community members’ perspectives, as per the request of community members.

### Data analysis

Transcripts were not returned to focus group participants due to confidentiality concerns; none of the individual interview participants were interested in reviewing their transcripts. In addition to research team discussions, individual transcripts were coded using reflexive thematic analysis, which seeks out patterns and formulates descriptive themes with the understanding that codes and themes reflect the researcher’s active role in interpreting the data [48]. We chose this analytic approach because it encourages a collaborative process of reflexive and thoughtful engagement with the data that, ideally, leads to a richer understanding of it. In our case, each transcript was read through once and initial topics of interest were highlighted and discussed by the lead researchers and community research assistants. We were not seeking “facts” and we less concerned with finding immediate consensus in our interpretations of the data; instead, we sought to explore different ways of understanding what participants felt was important to share with us in their own ways. Once the team was satisfied that recurring themes and patterns were identified, the two lead researchers did a second reading of each transcript to map out how coded lines with commonalities could be grouped under the same theme. Direct quotations were pulled from transcripts during a third reading to be included as examples of the codes. We further reviewed and confirmed these findings through conversations with our community researchers and partners.

### Ethics

We use Anishinaabe and Indigenous peoples here to refer collectively to the Ojibwe and Oji-Cree peoples living in northwestern Ontario, specifically in Robinson Superior Treaty and Treaty 9. We do not refer to specific First Nations or communities in order to ensure anonymity and confidentiality as requested by community members and required by Research Ethics. Conversations with community members and Elders guided the project to ensure that the research and research questions responded to concerns raised by community members and that our relationship was ethical and non-exploitative [49]. Participants gave informed consent prior to taking part in the study; the consulting process included information about the researchers and the purpose and the rationale of the study. Additional information regarding the ethical, cultural, and scientific considerations specific to inclusivity in global research is included in the S1 Checklist. This study received ethics approval from [Blinded for review] University REB #1465416.

### Inclusivity in global research

Additional information regarding the ethical, cultural, and scientific considerations specific to inclusivity in global research is included in the S1 Checklist.

### Findings

Participants expressed an eagerness to speak with us and to share their perspectives. Overall, participants clearly stated that they were not opposed to vaccination in principle, though past health care experiences sometimes influenced their attitudes. In some instances, their experiences left them with doubts about the underlying goals of immunizations and the purposes of vaccines. During our conversations with participants, we noted how the importance of trust and relationships (or lack thereof) affected participant attitudes toward immunizations and healthcare access and services.

Two themes were identified by participants as important to communicate with funders and fellow researchers: 1) trust relationships between rural and northern First Nations and the government, and 2) rapport and trust relationships between these communities and frontline health practitioners.

### Relationships with government service agencies

One point consistently made clear by participants was that Anishinaabe peoples saw their historical and ongoing experiences and relationship with the Canadian government health system and treatment by service providers as fundamentally different from non-Indigenous people. This made it difficult for them to trust that these government agencies had benevolent intentions. For example:

*It’s nothing for any white celebrity to go, well, I choose not to get my kid immunized, because that’s the way I’m going to do things as a parent. And then your kid goes to a private school, or you have them homeschooled, or whatever. And that’s fine, great choice for you. But I don’t have that choice, because my child would be taken away if I choose not to bring them to school. So, there are all kinds of things like that that—unfortunately or fortunately—non-Indigenous people don’t have to think about.*

*[FG 3]*

Thus, effective health care practitioners working with Anishinaabe populations must be aware of this double standard or lack of perceived choice and work to ensure that their encounters do not convey condescension, shaming, or coercion. An Anishinaabe health care worker made this very clear:

*I think what’s important is the way that you deliver the information, and to not come across as paternalistic or threatening by saying, ‘You have to do this,’ or wagging your finger and saying, ‘Your kids are really behind on their immunizations.’ You have to realize lots of them already feel bullied by state authorities, and that makes them less receptive to what you’re trying to tell them about the importance of immunizations.*

*[Practitioner 1]*

These quotations underscore how participants experienced their relationships with the settler government and health care service providers as lacking choice. People frequently described themselves as under scrutiny and living with the fear that any misstep might result in consequences that non-Indigenous people do not face.

When asked about their vaccination concerns beyond individual health matters, several participants described broader fears related to settler government institutions and the power the state exercised over Indigenous peoples. For example, some participants told us that they regarded vaccination schedules as a potential tool the schools could use to justify greater intrusion into their lives. Thus, a coercive relationship between vaccination schedules and schools was perceived to exist by many parents:

*Schools are problematic to our people…there’s a lot of judgment toward us that goes on there. It’s supposed to be a place of learning but often you feel like it’s a place of punishment when you don’t do as you’re told. Seeing a school nurse and her telling you what to do is part that. Vaccines are one more way, it feels like, that we are monitored by the government.*

*[FG 4]*

Several young parents further reported that the process by which schools notify parents that children require vaccines or risk suspension is often not constructive. According to respondents, this information is communicated in a manner that is often seen as aggressive, leaving people to feel as though they are being judged as negligent parents. Moreover, this approach felt coercive and robbed them of “choice” in health decision-making. According to a participant:

*They tell us they have to have their immunizations up-to-date, or they can’t be in school. I’ve heard the staff at the school even say, like, if kids aren’t vaccinated up-to-date then they can’t be there and we’ll call social workers or child services to come pick them up. So when all us became mothers, like, we always thought we had to do it. We didn’t know there was choice. We thought we had to get it done. We felt like we didn’t have no information and no say in what was happening with our babies.*

*[FG 6]*

Young parents also worried that a school suspension owing to incomplete vaccinations would lead to a child welfare agency becoming involved. In other words, parents feared that school officials would notify child services if their children were unvaccinated and, in turn, their children would be taken away. In the words of one participant:

*Participant: Well, I mean, especially for Indigenous people. If my son doesn’t go to school, the ‘law’ is going to come and check on me. They’re going to take my kid away, because, Why aren’t they going to school?*

*Interviewer: So the fear that these health agencies and schools work together to push vaccines in this way is a major concern for people?*

*Participant: Yes, that fear-mongering of the fact that someone’s going to come in and take your children, and blow your family apart, is definitely a high-pressure situation where you’re going to, well you’re just going to go with whatever you can to make sure your family stays together*.

*[FG 3]*

In short, participants’ attitudes toward vaccinations and healthcare, in general, were embedded in relationships of power, as reflected in their negative views of and historical and ongoing experiences with government institutions ranging from health clinics to residential schools, where many had received health care services, including immunizations. In their experience, settler government institutions are interconnected and coordinate surveillance and policing activities that have had and continue to have harmful effects on Anishinaabe peoples and communities, including child apprehension policies and practices.

Further adding to these negative perceptions was the common view that a double standard exists between the treatment of Indigenous and non-Indigenous peoples, which deprives them of decision-making power in their health care. Several health care providers noted that restocking health centers and nursing stations in First Nations, especially those communities that were fly-in access only for the majority of the year, was not a quick process and often took several weeks, if not months. In one interview, for example, a health care provider observed that they had regularly experienced the failure of vaccines to make it into fly-in First Nations due to poor weather conditions, noting “*the vaccines became useless after sitting on the tarmac for at least an hour in frigid temperatures before being placed on a plane” [Practitioner 10]*. Thus systemic differences in the quality of and access to health care that Anishinaabe peoples had also shaped the ability of people to “choose” immunization.

### Relationships with health practitioners

A second point often raised by participants, concerned having a bad rapport with health practitioners, a concern intimately related to trust. Several participants noted that prior “bad experiences” with practitioners had left them feeling unwilling and apprehensive about returning to complete immunization schedules:

*Some [health practitioners] are really rough with shots and then others are just, I don’t know, they don’t even show emotion or anything. They are just boom, boom, done, and go. So I don’t wanna go back, it’s a cold experience.*

*[FG 4]*

Another participant later added:

*Some [health practitioners] show no compassion or care for the children. They just shove the needle in, just really painful, kind of way harsh or they don’t try to distract them. It makes me not want to bring my kids back because it’s upsetting to me and then I have to deal with a child who’s upset and scared to go to the nurse again.*

*[FG 1]*

Even participants who recognized the value of immunizations reported feeling deterred from seeking these services following a negative experience with a service provider. Further, some parents with more than one child added that after a bad immunization experience with one child, they would avoid bringing their other children for health services.

The health practitioners we spoke to, who closely work with Anishinaabe communities, shared similar stories while explaining how they attempt to avoid these practices by establishing trust with patients.

*The importance of something like vaccines is not always on their radar, even if they know it. You know, I try to prepare them in a nice way to get their babies vaccinated. See, if they feel someone was rough at the two-month visit, they will avoid coming back for the four-month visit. You have to meet people where they’re at; a bit of empathy can go a long way with people.*

[*Practitioner 2*]

*Really, I think the [vaccination] uptake also depends on their relationship with the provider. So if they trust you and you’ve built that trust relationship, they’re more willing to listen when you say, ‘This is important and here’s why,’ and usually they’ll say, yeah, no, that makes sense, yeah, let’s do that*.

*[Practitioner 7]*

In short, the testimonies highlight the importance of a positive vaccine experience to maintain relationships, trust, and continued vaccinations. Health care service providers who work closely with Anishinaabe communities and are familiar with their feelings towards government agencies noted the importance of going the “extra mile” to communicate medical information and address people’s concerns proactively and respectfully.

## Discussion

Government agencies and the practitioners working within them are the mechanisms through which healthcare is delivered; therefore, trust and rapport with these entities are paramount for vaccine uptake and healthcare efficacy. Our findings highlight the importance of trust relationships between communities and health practitioners and that these relationships cannot be understood separately from historical and ongoing encounters between Anishinaabe peoples and the settler government. Notably, the findings illuminate how Indigenous community members perceive their relationships with social and health services and education as connected and lacking choice. The lack of trust ultimately leaves some parents questioning the purpose of vaccines to prevent illnesses. Respondents often expressed fears that child welfare agencies would apprehend their children if they were unvaccinated. Specifically, parents feared school officials or others in the community could report them to child welfare agencies. These fears are borne out of both personal experience and shared knowledge. Milne and Wotherspoon (2020, p. 36) convey that “…for many Indigenous families, it is an ongoing reality, not simply a relic of a colonial past, that school sites contribute to fears about child displacement” [50]. For instance, a 2018 report revealed that Indigenous children in Ontario represent at least 30% of children in care, despite only constituting 4.1% of the total population under the age of 15 [51]. In northern regions of the country, this proportion is much higher.

Although Indigenous peoples access a range of health and social services out of necessity, they are well attuned to the potential for danger, whereby these agencies function as mediators between families and the state [50]. To participants, the nature of relationships with health and social services was deeply intertwined with Indian Residential Schools, a school system operated by the Canadian government from 1867 to 1996, when the last federally operated school was closed [52]. Residential schools were established to forcibly remove Indigenous children from their families and communities and place them in government-funded church-run schools designated to “eliminate parental involvement in the intellectual, cultural, and spiritual development of Indigenous children” [52]. This association leaves some parents questioning if the real purpose of vaccination schedules is an excuse to surveil and further police Indigenous communities. Participants were very clear that they wanted access to healthcare and were not opposed to vaccines in principle. Still, they wanted ease of access to good and reliable services, such as care that is consistently culturally appropriate and anti-racist. People wanted to be treated with dignity and respect (their words), two elements intimately connected to trust and rapport between patients and practitioners.

In much of northern Ontario, particularly in communities without all-season road access, people receive healthcare via community health clinics or nursing stations. Health care workers in these spaces are frequently transitory, overworked, and arrive with very little foresight regarding the job demands [53], which may help explain why participants reported interactions between practitioners and community members tend to be fraught and difficult. Several young parents reported that health care workers had treated their children “roughly” when providing services or that providers presented as uncaring when children cried while receiving injections. Again, the negative relationship made young parents hesitant to revisit these service providers, thus making it less likely that they would learn about vaccination requirements or receive continuity of care moving forward.

There is literature that speaks to the deleterious consequences that negative experiences with health practitioners have on vaccine uptake and healthcare more generally [54–57]. For Indigenous peoples, however, these experiences must be considered within a broader context of settler colonialism and state violence. It is not just about one vaccine but includes long histories of medical experimentation on Indigenous bodies and the removal of people, especially children, from their communities for treatment without consent [58–60]. Thus, when considering how to implement and inform immunization policies, we cannot formulate them in isolation from these histories and experiences. For many Anishinaabe peoples, their negative experiences with health practitioners cannot be read as isolated incidents but need to be understood as part of a broader pattern and history affecting their families’ and communities’ health and well-being.

Duly, these findings require that we return to our earlier question: is vaccine hesitancy the best term for describing Indigenous peoples who delay or do not get vaccinated? Among our participants, decisions to accept, delay, or reject vaccines are partly mediated by historical and ongoing experiences with racism and colonialism. Indeed, our findings indicate that a problematic relationship with government and health welfare agencies compounded by broken or underdeveloped trust relationships between health practitioners and Anishinaabe communities underlie much of the vaccine resistant sentiment we have identified thus far. Use of the blanket term vaccine hesitancy can convolute these distinct ongoing colonial histories and instead place blame on Indigenous peoples and communities [24], problematizing them as irresponsible and risky [9]. In doing so, public health measures continue the colonial legacy by functioning as a tool of control for the settler state [9]. For example, a settler Canadian adult who does not have the same history and experience of forced sterilization, medical experimentation and health care providers being “rough” with their children may be more likely to vaccinate themselves and their child(ren), earning them the titles of “good citizen” and “good parent” while an Indigenous adult who delays or refuses the vaccination is labelled as irresponsible, risky, and a “bad parent,” providing grounds for state intervention and control over their life.

Furthermore, acting upon our commitment to stop “damage-centered research” [44] we must also acknowledge the need for a conversation that goes beyond getting Indigenous peoples to say “yes” and supports the idea that Indigenous communities have “their own wisdoms and teachings” that can complement and support public health strategies for addressing infectious and communicable diseases [10]. Damage centred research focuses on identifying the supposed deficits within Indigenous communities and bodies rather than locating problems in systems of white supremacy and colonialism [44]. Within the current framing of vaccine hesitancy, Western society and science is represented as the progenitor of innovation, technology and all solutions to global problems [61]. This latter approach places Indigenous and Western knowledge in competition with each other rather than as complementary and supportive. Instead of vaccine hesitancy, Greenwood and MacDonald use the term *vaccine mistrust* to highlight the importance of Indigenous knowledge and wisdom and the centrality of personal choice [10]. Unlike vaccine hesitancy, this term can co-exist within a public health framework that acknowledges and respects Indigenous peoples’ right to self-determination whereby vaccine mistrust can be addressed by a spectrum of approaches, including improving health care provider and social service agency relationships with Indigenous peoples, culturally and community driven approaches to immunization, and the use of Indigenous healing systems. We suggest drawing on a two-eyed seeing as a guiding principle for moving forward where “we see from one eye with the strengths of (or best in) Indigenous knowledges and ways of knowing, and learning to see from the other eye with the strengths of (or best in) Western knowledges and ways of knowing …and, most importantly, using both of these eyes together for the benefit of all” [62].

### Strengths and limitations

These observations may be unique to this region based on its specific historical and ongoing colonial context and experiences. Moreover, Indigenous communities are heterogeneous regarding knowledge, attitudes, and beliefs. Our focus group sample was skewed in favor of female participants. This was perhaps due to cultural reasons, with women the most likely to manage medical appointments and provide healthcare, particularly where children are concerned. Despite these limitations, to date we found consistent experiences and concerns among participants. This study benefits from working with community-based research assistants who are trusted by community members and from our ability to rely upon the community relationships we have developed in this region over many years. These relationships enabled us to recruit a relatively diverse group of participants and helped to alleviate feelings of distrust held by community members. However, our efforts to recruit young parents and elders for their views on vaccines may have resulted in an overrepresentation of people who are inclined to favor vaccines due to perceptions of risk.

Another limitation of this study is that it worked solely within a Western health paradigm. Specifically, the intention of the study was to understand barriers to vaccination as a step toward improving vaccination rates. This may have hindered the ability for other insights to emerge that can support culturally safe approaches to public health planning such as the use of traditional medicine in conjunction with immunization or as an alternative to vaccination.

## Conclusion

Initially, this study aimed to explore Hia; however, only some people we spoke to knew about this disease. Instead, people wanted to have discussions about trust, respect, and rapport concerning vaccines and immunizations, reflecting their negative experiences with the state’s power over them. What became clear from these conversations is that unless these three issues are adequately addressed, no immunization policy would be welcomed and relationships with health care providers cannot be productive.

Repairing relationships with Indigenous communities and investigating the possibility of regular community health care service providers may be one path forward and has been identified by the Truth and Reconciliation in their Calls to Action [52]. Our fieldwork indicated that people with access to regular and reliable service providers were more comfortable with vaccines and trusting vaccination schedules, which is also supported in previous studies [63–65]. This held true when service providers were private practitioners or public health nurses. Therefore, repairing and building trust relationships stands in contrast, for example, to simply providing information pamphlets about vaccines, holding temporary immunization clinics staffed by transient service providers, or using child welfare to ensure immunization compliance.

Public health approaches cannot continue to operate in ways that prioritize interventions for Indigenous peoples and communities so that they “do the right thing.” More emphasis is needed on health service and social service provider knowledge, skills, attitudes and practices-redirecting the onus onto those within the health care system. Drawing from participant responses and lessons learned from COVID-19, a responsible and respectful approach regarding Hia vaccination entails the following: rebuilding trust through respectful dialogue and empathy [66], engaging with communities about Hia disease [22], acknowledging the legacy of mistrust, supporting community-driven implementation of a new Hia vaccine program [67], drawing on the strengths of Indigenous culture, beliefs, community and knowledge systems [10, 16, 67], ensuring consistent and accessible services [66], and respecting Indigenous peoples’ right to be self-determining in regard to health and governance [10].

As noted, these approaches have already proven to be successful during the COVID-19 pandemic. For instance, Anishinaabe leaders and Elders worked extremely hard to address community members’ vaccine concerns. Anishinaabe leaders used social media, drew on community relationships and attended vaccine clinics in fly-in communities, often stepping up to be the first vaccinated to show by example or speak with community members when they were concerned [68]. In another instance, MPP Sol Mamakwa created videos in Oji-Cree specifically designed to encourage community members to get vaccinated. A social media campaign encouraging youth to create their own videos incentivized youth to get vaccinated and engage with the issue more broadly [69]. The COVID-19 vaccine work undertaken by Anishinaabe leaders and Elders in the region offers a model for non-Indigenous leaders and health care practitioners regarding effective and respectful collaboration [16]. It highlights Indigenous self-determination and leadership as a necessary foundation for any Indigenous public health planning.

## Supporting information

S1 Questionnaire Interview guide. (DOCX)

S1 Checklist COREQ (COnsolidated criteria for REporting Qualitative research) checklist. (PDF)
